# Getting Active with Active Video Games: A Quasi-Experimental Study

**DOI:** 10.3390/ijerph17217984

**Published:** 2020-10-30

**Authors:** Yan Liang, Patrick W. C. Lau, Yannan Jiang, Ralph Maddison

**Affiliations:** 1 Department of Physical Education, China Women’s University, Beijing 100101, China; liangy@cwu.edu.cn; 2 Department of Sport, Physical Education and Health, Hong Kong Baptist University, Hong Kong, China; 3 National Institute for Health Innovation, University of Auckland, 1010 Auckland, New Zealand; y.jiang@auckland.ac.nz; 4 Institute for Physical Activity and Nutrition, School of Exercise and Nutrition Sciences, Deakin University, Melbourne, VIC 3125, Australia; ralph.maddison@deakin.edu.au

**Keywords:** children, sedentary time, physical activity, active video game, accelerometer, after-school time

## Abstract

Given the cultural emphasis on academic achievement and environmental constraints to physical activity (PA), active video games (AVGs) may be used to decrease sedentary behavior and increase PA of Hong Kong children. This study explored the potential of a school-based AVG intervention on sedentary time, PA, body composition, and psychosocial factors among children. Eighty-seven children (intervention *n* = 30) were recruited from one primary school. Classes in Grades 4–6 were allocated to either the intervention group or the control group in a 2:1 ratio. The eight-week intervention involved children playing AVGs in an after-school class twice a week. Participants in the control group continued with their usual activities. Outcome included the change of participants in sedentary time, PA, percentage body fat, body mass index (BMI), and psychosocial variables (enjoyment, self-efficacy and social support), from baseline to eight weeks. No significant group differences were observed in sedentary time (−33.9 min/day, 95% CI −70.8 to 4.8; *p* = 0.07). The intervention group significantly increased total PA (53.7 counts/min, 95% CI 8.6 to 104.2; *p* = 0.04) compared with those in the control condition. No differences were found in body composition and psychosocial variables. However, significant treatment effects were found on BMI z score among boys (−0.1, 95% CI −0.2 to 0; *p* = 0.04). An eight-week school-based AVG intervention delivered during after-school hours was effective in increasing activity levels among Hong Kong children. The treatment effects of AVGs on sedentary behavior and body composition need to be further demonstrated in a more robust study, especially in boys.

## 1. Introduction

Children are widely recommended to engage in at least 60 min of moderate-to-vigorous physical activity (MVPA) every day [1,2], and decrease sedentary screen activities to at most 2 h per day [3] for health. The lack of physical activity (PA) contributes to childhood obesity and other health-related negative consequences [4]. However, a large proportion of Hong Kong primary school children did not achieve these PA recommendations [5]. This may result in the high prevalence of overweight/obesity among this population [6]. Furthermore, the rates of overweight/obesity were consistently higher in boys than in girls in the last decade [7]. These data call for effective strategies to increase PA levels and prevent obesity among primary school children in Hong Kong, especially in boys.

It is well known that time spent outdoors is positively associated with the PA levels of children [8]. The problem in Hong Kong is the limited outdoor space in primary schools (standard is 2 m^2^ per student) [9]. Furthermore, given the constraints of outdoor programs (i.e., safety concern, air pollution, and inclement weather), indoor programs may be an option to increase activity levels. Potential components of such programs may include delivery of active lessons and activity breaks; however, these strategies may not be sufficiently intense to result in MVPA [10]. The implementation of these strategies likewise requires considerable resources to train classroom teachers and alter the classroom environment. Therefore, novel strategies are warranted to be examined.

Active video game (AVG) technology incorporates body movements into videogame play, and may provide an attractive option for displacing sedentary screen time and increasing PA in children. One systematic review [11] extracted child specific metabolic equivalent of task (MET) values (dividing oxygen uptake during AVG play by resting oxygen uptake) for AVGs, and AVG intensities were considered as light (<3 METs), moderate (3–6 METs), or vigorous (>6 METs). Within the laboratory, certain AVGs have been shown to elicit moderate-intensity activity among children [11]. Using age-specific cut-off points of accelerometer counts, Norman et al., also found that in the free-living environment, some AVGs could reach moderate intensity [12]. These studies supported that AVG could be used as a possible indoor intervention strategy to increase PA levels. 

In the past decade, there has been increased research interest in the use of AVGs to promote PA and curb childhood obesity. AVGs have been used in home, school, community, and healthcare settings. Most trials conducted within the home setting have shown that AVGs do not positively influence objectively measured PA and sedentary behavior of children [13,14,15,16,17,18]. One potential explanation is that children did not play AVGs during the whole intervention period, thus representing low intervention fidelity. In one study, the median self-reported playing time of a dancing game decreased from 228 min in the first 6 weeks to 0 min in weeks 7–12 [19].

Previous free-living studies have mainly focused on children’s individual play. Several studies have showed that when play was organized by school teachers or researchers in a structured way, instead of free play at home, there was an increase in PA levels [11,20]. However, the evidence needs to be strengthened in the school-based AVG interventions [11]. No study has jointly measured habitual PA and sedentary time by the objective way like the family-based studies. Therefore, it is still not clear whether the school-based AVG play could influence children’s habitual PA and sedentary time. 

To improve our understanding of the effectiveness of AVG interventions on PA behavior, it is also important to understand potential mediating and moderating factors. In existing school-based AVG interventions, two studies [20,21] jointly measured PA and PA related psychosocial factors. Such research may inform the potential mechanisms of structured AVGs on PA. The existing results were equivocal. Lau et al., found that AVGs could increase children’s PA but had no effect on PA enjoyment and self-efficacy [20]. However, Gao et al., found that playing AVGs at school could increase children’s exercise efficacy and social support [21]. Therefore, enjoyment, self-efficacy, and social support were selected to be further examined in the present study. The current study aimed to determine the effects of a school-based AVG intervention on sedentary time, PA, body composition, and psychosocial factors among Hong Kong children. When significant group differences in sedentary time and PA outcomes were found, this study also determined to examine the mediation and moderation effects of psychosocial factors within the intervention.

## 2. Materials and Methods 

This study was approved by the Committee on the Use of Human and Animal Subjects in Teaching and Research, Hong Kong Baptist University (Ethical Code #:HASC/Student/12-13/003).

### 2.1. Participants

Participants were recruited from one Hong Kong Government primary school in the New Territories of Hong Kong. Children were eligible to participate in the study if they (1) were in Grades 4–6 (9–12 years old); (2) were free from physical or psychological constraints to being active; (3) did not participate in any school sports team; and (4) did not participate in an extracurricular exercise class. Ethical approval and written informed consents provided by both participants and their guardians were obtained prior to data collection. 

**Figure 1:**
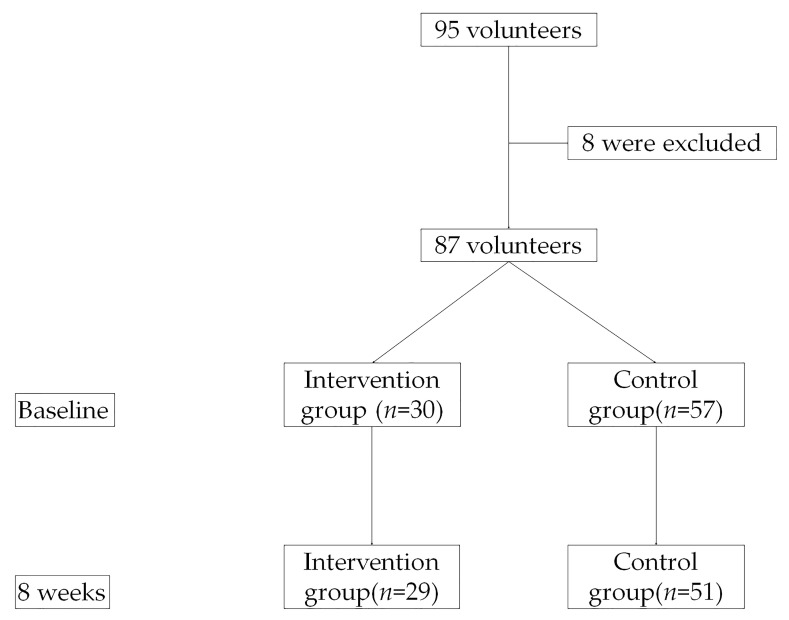
Flow Chart of Participants through the Study.

A total of 95 children volunteered to participate in the study, of which eight children were excluded due to eligibility. For the 87 study participants, 30 children (24 males) were in the intervention group and 57 children (30 males) were in the control group. One girl in the intervention group dropped out because of time constraints with extracurricular academic activities. Six children in the control group did not provide data at 8 weeks. A flow chart of participants through the study was presented in Figure 1.

### 2.2. Design

A quasi-experimental design was used to explore the acceptance of children and their guardians to the AVG intervention. Classes in Grades 4–6 were allocated to either the intervention group or the control group in a 2:1 ratio, stratified by grade. Children in the intervention classes were provided an after-school extracurricular AVG class option, which was one of the options they could choose for after-school activities organized at school. The control classes were not provided this after-school option.

#### 2.2.1. Intervention

The intervention group attended the after-school AVG classes for 8 weeks, with two 1 h (3:00 p.m.–4:00 p.m.) sessions a week. Eight weeks is a minimal and feasible duration to produce impact on children’s objectively measured PA under the school schedule constraints [22]. 

At the beginning of each 60 min session, participants underwent a 10–15 min warm-up (led by one of the authors or a research assistant). During this time, research assistants (trained graduate students) set up the classrooms for playing AVGs. Two game consoles-Xbox 360 Kinect^TM^ (Microsoft, Redmond, WA, USA), which supported multiplayer use (2–4 children can play AVGs simultaneously), were connected to a TV set and a projector in each classroom. To encourage children to play AVGs, at most two pairs of children played AVGs in each classroom. Therefore, children did not need to wait for their turns to play AVGs. Children themselves selected their preferred playing partner. This approach was designed to enhance the perceived social support of the participants. The children who played AVGs on the TV would play AVGs on the projector in the next class. Children were allowed to take short breaks during AVG classes. After 4:00 p.m., the intervention children stopped playing and left school [23]. 

In the first 4 weeks, Kinect “Adventures” (Good Science Studio, Microsoft Game Studios), which consists of five games (“20, 000 Leaks”, “River Rush”, “Rally Ball”, “Reflex Ridge”, and “Space Pop”), was used. These games require both upper and lower body movements. “Adventures” has been shown to elicit moderate intensity PA among children (METs = 4.4) [24]. The participants could freely choose from the games to enhance their intrinsic motivation [25]. In the next four weeks, children were able to choose either Season 1 or Season 2 of Kinect “Sports” (Rare, Microsoft Games Studios). Each season of Kinect “Sports” consists of six sports simulation games. Of the games available in Kinect “Sports”, boxing has been shown to elicit moderate intensity activity (METs = 4.0) [26]. These sport games were provided to keep the interest of the participants [23].

#### 2.2.2. Control

The control participants engaged in their usual activities during the intervention periods, either leaving school or participating in non-exercise-based extracurricular activities at school during the intervention period [23].

### 2.3. Procedure

Inclusion criteria were assessed using a screening questionnaire and confirmed by schoolteachers. Assessments were conducted by trained researchers at the end of the school day for both pre- and post-test. The participants received individualized health recommendations in sealed envelopes regarding their PA levels and body composition after all assessments to promote their compliance.

The participants attended a briefing on the measurement of accelerometry before the baseline assessment. Children were instructed to wear the accelerometer on their right hip with an elastic belt during waking hours, except sleeping, bathing, and doing water activities and contact sports. A wearing log with written information was provided after the briefing. Participants recorded wearing details, such as when they put on and removed the accelerometer, and the reason for each removal (e.g., swimming). The log was used to encourage the participants to wear the device; however, it was not used to input non-wear time. Intervention children who attended at least 80% of the sessions were reimbursed for extra school bus fees incurred for attending AVG classes [23].

### 2.4. Measures

#### 2.4.1. Anthropometrics

Standing height was measured twice to the nearest 0.1 cm using a portable stadiometer (Seca, Model 214, Hamburg, Germany). Weight (measured to the nearest 0.1 kg) and percentage body fat were assessed using a bioelectrical impedance analyzer (Tanita, Model TBF-410GS, Tokyo, Japan) [23]. This analyzer has demonstrated validity to assess percentage body fat among Hong Kong Chinese children [27]. Body mass index (BMI) was computed by weight (kg) divided by height squared (m^2^); zBMI was computed as recommended by Cole and Lobstein [28].

#### 2.4.2. Physical Activity and Sedentary Behavior

Objectively measured PA and sedentary behavior were assessed using the GT3X and GT3X+ accelerometers (ActiGraph, Pensacola, FL, USA). For each outcome assessment, participants were asked to wear the accelerometer for seven consecutive days. Because this study was a school-based intervention, only school-day data were analyzed. 

Activity counts were collected in 5 s epochs, and integrated to 1 min intervals. Only the data recorded between 7:00 a.m. and 10:00 p.m. were considered as waking time due to various sleep patterns [29]. Non-wearing time was defined as 60 min or more of consecutive zero records. A valid day included 480 min or more of wearing time. At least two valid days of data were required for inclusion [30]. In addition, data were further reduced from 3:00 p.m. to 10:00 p.m. in order to determine the treatment effect during the targeted after-school time. The participants who provided at least two days of after-school time data (60 min or more of wearing time for one day) were included for the additional analyses [23].

Time spent in sedentary behavior and different intensities of PA were derived based on established cut-off points of counts [31], which have been demonstrated to estimate different intensities of PA in the targeted age group [32]. The participants at baseline reported sedentary screen behaviors with a questionnaire previously used in this population [33]. 

#### 2.4.3. Psychosocial Factors

Enjoyment of PA was assessed using a 7-item scale (e.g., “when I am active, it’s not at all interesting”) ranging from 1 (“disagree a lot”) to 5 (“agree a lot”) [34]. Ten items (e.g., “during the past three months, my family did PA with me”) adapted from the social support for exercise scale [35] were used to measure social support for PA. Participants rated how often (1 = none to 5 = very often) they received social support from family and friends, respectively. An 8-item scale (e.g., “I can do active things because I know how to do them”) was used to measure PA self-efficacy ranging from 1 (“disagree a lot”) to 5 (“agree a lot”) [36]. This scale was adapted based on two published versions [34,36]. Ratings of each scale were averaged. All of the used items have been translated to Chinese and supported to be used in Hong Kong Chinese children [37].

#### 2.4.4. Process Evaluation

Attendance for the AVG classes was recorded, and the intervention participants were interviewed after the intervention to rate the class from 1 to 5 (higher is better).

### 2.5. Outcomes

Outcomes of this study included changes in daily sedentary time, MVPA, light PA (LPA), moderate PA (MPA), vigorous PA (VPA), counts per minute (CPM), after-school sedentary behavior and PA, BMI z score, percentage body fat (%), and PA related psychosocial factors (including enjoyment, self-efficacy, and social support) from baseline to 8 weeks.

### 2.6. Statistical Analyses

All statistical analyses were conducted using IBM SPSS Statistics version 21.0. Statistical tests were set at a two-tailed 5% significance level. Descriptive statistics were used to summarize all measures of interest at baseline by treatment group. Continuous variables were presented as mean and standard deviation, and tested using independent *t*-test between groups. Categorical variables were presented as frequency and percentage, and tested using Chi-square test between groups. Linear regression analyses were conducted on outcomes adjusting for important baseline prognostic factors. For sedentary time and PA outcomes, the models were controlled for baseline outcome value, age, gender, zBMI, and average daily wearing time. For body composition outcomes, the models were controlled for baseline outcome value, age and gender. For psychosocial outcomes, the models were controlled for baseline outcome value, age, gender and zBMI [23].

Model assumptions were tested as necessary. Multi collinearity was checked by the Pearson correlation coefficients between the continuous independent variables, and the variance inflation factor. The overall model significance was assessed using the F-test in analysis of variance (ANOVA), and individual *t*-tests were used to test the predictive effect of independent variables in the model. Normality of the continuous outcome variables were checked using K-S test. The bias corrected and accelerated bootstrapped confidence intervals (BCa CI) of the group differences based on 1000 samples were obtained in order to reduce bias. The missing data were excluded from analyses. Adjusted group means and standard errors, group difference with associated 95% BCa CI and p value in each outcome were reported [23].

Potential mediation and moderation effects of psychosocial factors were examined using hierarchical regression models when treatment effects found in sedentary time and PA outcomes [38]. For mediation analyses, the change score of each psychosocial factor (i.e., enjoyment, self-efficacy, social support from friends and social support from family) from baseline to 8 weeks, plus the interaction term with the group variable, were added to the regression models for estimating the treatment effects. If the main effect or the interaction effect of one psychosocial variable was significant, it was considered as a mediator of the treatment effect. Similarly, the baseline measure of each psychosocial factor was added to the regression models plus its interaction term with the group variable. If the interaction effect was significant, the psychosocial variable was considered as a moderator. 

Given that the intervention participants were predominantly male students (80%) and the gender differences in activity levels and PA correlates [39], sub-analyses were then conducted for boys (*n* = 54) to evaluate the consistency of main findings obtained from all participants.

## 3. Results

### 3.1. Baseline Characteristics of All Participants

Demographics of 87 participants were presented in Table 1. No group differences were found at baseline in age, height, weight and BMI. More male participants were in the intervention group than in the control group. Children reported that they engaged in more than two hours in sedentary screen behaviors, including TV viewing, video game play and computer use. There were no differences between the intervention group and the control group in sedentary screen time.

### 3.2. Main Findings Obtained from All Participants

The mean changes from baseline to 8 weeks in daily time spent in sedentary behavior were −30.1 min and 3.8 min in the intervention and control groups respectively (Table 2). However, the group difference of −33.9 min was not statistically significant (95% BCa CI −70.8 to 4.8, *p* = 0.07). No significant group differences were observed for the change in average daily time spent in MPA (3.4 min, 95% BCa CI −0.3 to 7.3; *p* = 0.06). Significant differences were found for the change in LPA (34.9 min, 95% BCa CI 8.7 to 58.2; *p* = 0.01), and CPM (53.7, 95% BCa CI 8.6 to 104.2; *p* = 0.04), but not for VPA. 

During after-school time, a significant treatment effect occurred on sedentary time (−23.5 min, 95% BCa CI −41.7 to −5.4; *p* = 0.01), LPA (25.0 min, 95% BCa CI 13.7 to 34.5; *p* < 0.01), and CPM (109.4, 95% BCa CI 36.4 to 178.8; *p* = 0.01). The group difference of change in MVPA from baseline was not significant (3.0 min, 95% BCa CI 0.2 to 5.9; *p* = 0.07). Similar trend was obtained for MPA (2.4 min, 95% BCa CI −0.2 to 5.1; *p* = 0.08), but not for VPA. 

No significant group differences were found in zBMI, or percentage body fat at 8 weeks (Table 3). No significant group differences were found in any of the measured PA-related psychosocial factors, specifically in enjoyment, self-efficacy, social support from friends, and social support from family (Table 4).

### 3.3. Mediating and Moderating Analyses

Changes of LPA and CPM from baseline to 8 weeks were used as the dependent variables in respective regression models to examine the mediation and moderation effects. None of the PA-related psychosocial factors met the criteria for mediators or moderators. Changes of sedentary time, LPA and CPM during after-school time were also used as dependent variables to test regression models, neither mediation nor moderation effects of PA-related psychosocial factors were noted as well.

### 3.4. Sub-Analyses for Male Participants

Among the male participants, no group differences were noted at baseline. Treatment effects at 8 weeks were reported in Table 5. In general, these results were similar with those obtained from all participants. However, significant treatment effects were found on BMI z score among boys at 8 weeks (−0.1, 95% BCa CI −0.2 to 0; *p* = 0.04).

### 3.5. Process Evaluation

Average attendance of the intervention was 91.0%. At 8 weeks, 38% of the validated days to estimate the waking hours PA of intervention participants were with intervention sessions, and 35% of the validated days to estimate the after-school hours PA of intervention participants were with intervention sessions. These data were consistent with the frequency of the AVG classes (40% of the school days). The participants rated the AVG class at an average of 3.6.

## 4. Discussion

This study aimed to explore the potential of a school-based AVG intervention on sedentary time, PA, body composition, and psychosocial factors among Hong Kong children. This study is among the first to determine the school-based AVG intervention effect on children’s overall sedentary time. The results showed that a school-based AVG intervention delivered twice a week (in total 120 min session weekly) decreased children’s sedentary time 117.4 min/week and increased LPA 125 min/week during after-school hours at Week Eight. Average attendance of the intervention was 91.0%. The intervention ratings of participants were positive (3.6 out of 5). These results together with the treatment effects are promising to show that the intervention could be well accepted by inactive and low sport competence children (results indicated participating children engaged in only one third of the recommended MVPA level), and encourage them to get active. The methods of the present AVG intervention are practical and feasible for use in other Hong Kong primary schools.

The observed daily sedentary time of Hong Kong children was about 8 h per day in the present study, and 3 h of sedentary behaviors occurred in the after-school time (3:00 p.m. to 10:00 p.m.). Types of sedentary behaviors could not be identified using accelerometry. Therefore, a questionnaire was used to assess children’s sedentary screen time. Results suggested that Hong Kong primary school children engaged in more than two hours in TV viewing, video game play and computer use. This result was consistent with previous data using the same questionnaire [34]. Given the emphasis on the academic and examination achievement in the Chinese culture, children are highly occupied by the tutoring classes after school [40]. This phenomenon lead into significant sedentary behavior of Chinese children [41]. The present study indicated that sedentary time was reduced when they engaged in AVG. It is highly possible that children replaced their sedentary screen time to play AVGs instead of decreasing their sedentary time doing homework or attending extracurricular academic classes. For this population, the decreasing 30 min of daily sedentary time may result in a promising effect on health related outcomes, such as fitness [20]. This finding may provide more reasoning for schools and parents to decide whether to use AVGs to promote children’s PA and health. The positive attitudes of relevant stakeholders, including school principals, teachers, parents, and participants towards school-based PA interventions are crucial for the implementation [23]. Given that the increasing evidence on the independent health effects (including physical, emotional and social health indicators) of reducing sedentary behavior in children [42,43], the comprehensive health effects of AVG interventions among children is warranted to be further studied. In addition, the treatment effect of a school-based AVG intervention on children’s sedentary behavior needs to be further examined in a larger trial.

Children increased their habitual PA level (53.7 counts/min) after 8 weeks of AVG play during the after-school sessions. Results suggested the increased PA were mainly in the light intensity (34.9 min/day, *p* = 0.01), but not in moderate-to-vigorous intensity (3.6 min/day, *p* = 0.12). In addition, the increased LPA was in consistence with the decreased sedentary time. This result was consistent with the previous literature that children played most AVGs in the light intensity [11]. Children who played AVGs in the after-school time increased their LPA 25.0 min/day (*p* < 0.01) during after-school hours, and further increased their LPA level in other time, instead of decreasing their PA level to compensate the more energy expenditure. Evidence has been accumulated on the health effects of LPA among children, such as decreasing adiposity [44] and cardiometabolic biomarkers [45], especially when LPA replacing sedentary time. However, the research focused on the LPA of children was not as much as the one studying MVPA. Along the same line, previous field-based AVG interventions mainly reported intervention effects on MVPA [11]. This may result in the underestimate of the influence of AVG play on children’s PA. 

A randomized controlled trial conducted in Hong Kong found that children who also played Xbox Kinect twice a week (each session for 60 min in duration) increased their MVPA 6.73 min/day compared with the control group [20]. There were several differences between the present study and the previous study [20], including the shorter play session (a 10–15 min warm-up session should be deducted from the total 60 min session in the present study) and the smaller group size of children who played AVGs together resulting from the smaller play space of the present study. These differences may explain the different results in MVPA. In addition, both studies recruited children only from one school in Hong Kong and the sample sizes were no more than 100. These facts limited the generalization of the results to children in other schools. To what extent and how a school-based AVG intervention could influence children’s MVPA still need to be studied in a larger scope in Hong Kong.

The observed treatment effect on zBMI among the male participants is along the line of the observed increased PA level (energy expenditure). Other than energy expenditure, the replacing effect of AVG play on the sedentary behavior may also influence the energy intake side. Studies found that sedentary screen time were associated with higher intake of energy-dense snakes and sugar-sweetened beverages [46]. The treatment effect of school-based AVG play on children’s BMI should be further studied with extended duration and a more robust study design, especially in boys.

The reason that the null effect of the AVG intervention on zBMI among all participants was noted may be due to the gender differences in the weight status, sedentary behaviors and video game interest. The Hong Kong boys are facing a more serious problem in obesity than girls. Staiano et al. [47] observed that the structured AVG play as a single component could produce body weight loss among overweight/obese adolescents over 20 weeks. In the home setting, Maddison et al. [15] determined a small effect of home-based AVG play over 24 weeks on BMI and body composition among overweight/obese children. Trost et al. [48] found that integrating AVGs into a family-based weight management program over 16 weeks had a positive effect on body composition among overweight/obese children. In the contrast, a nine-month school-based intervention conducted in 185 general primary school children found no effect on body composition [49]. Collectively, these studies suggest that the baseline weight status may moderate the effects of AVG interventions on children’s weight. 

The present study and a previously published study [20] both found that boys are more likely to participate in the AVG intervention in Hong Kong, which reflected the greater use and interest of video game by boys in reality [50]. In addition, previous literature showed that boys played AVGs (the same games as the ones in the present study) more intensively than girls [26]. Therefore, researchers and other stakeholders should consider this gender differences when they use AVGs to increase PA or decrease adiposity in the primary school students, given that the novel strategy may not be well accepted by the girls and their guardians (80% vs. 53% of participants in the AVG intervention group and the control group respectively were boys). 

The null effect on percentage body fat suggested that the 8-week AVG intervention may not be long and intensive enough to induce change in body composition in the general children population. However, based on the found treatment effect on BMI z scores among boys, AVGs could be considered as a useful tool to decrease adiposity among overweigh/obese children in Hong Kong, especially for boys. 

The null effect of the AVG intervention on enjoyment of PA is consistent with the study of Lau and his colleagues [20]. It may be partially due to the simulated sports (e.g., boxing, baseball) were not popular in Hong Kong Chinese children. Cultural specific AVGs should be considered in the future studies, if we want to induce more health benefits. Games can be matched to children’s sport interest, language, age and gender. Although children indicated that they enjoyed AVG play in previous laboratory-based studies [51,52], the acute affective effect of AVG paly may not be transferred to the general PA as we measured in the present study. In addition, we used a negatively worded PA enjoyment scale [37] in the present study (e.g., “when I am active, it’s not at all interesting”), which may influence children’s answers. Therefore, negative wording questions should be avoided in the future studies in young children. 

Two previous studies [21,47] found favorable intervention effects of structured AVG play on exercise self-efficacy. In contrast, no treatment effect was found on PA self-efficacy in the present study, which was consistent with the previous Hong Kong study [20]. The different AVGs used in the studies may partially explain the discrepancy. The AVGs played in the present study were easy for children to play, and there were no difficulty levels for them to choose as a dancing game used in the previous study [21]. Children may increase their efficacy when they level up the game play, therefore lack of difficulty levels in the present study may not encourage children’s self-efficacy. In addition, there is a gap between the virtual world and the real world. Therefore, even children who played simulated sports well, the experiences might not be transferred into the real world sports and consecutively increase their self-efficacy. The mixed evidence of AVG play on PA related self-efficacy calls for further study to clarify to what extent and how structured AVG play may enhance children’s self-efficacy. 

In contrast to the present study, a 9-month AVG intervention [21] found a treatment effect on exercise related social support (comprised of parental, peer and teacher support). A 20-week AVG intervention [47] found that children who played AVGs every day at school increased their general peer support. Compared to the two studies [21,47], the duration of the school-based AVG interventions in Hong Kong (the present study and the previous study [20]) were much shorter. The influence of school-based AVG intervention on psychosocial factors may need longer time to occur. Usually, children’s interactions are not encouraged in classrooms in Hong Kong schools to form a quiet learning environment. For this reason, the participating children in the present study may not talk or interact as much as the ones in the previous studies conducted in USA [21,47].

Cooperative AVG play between partners could induce favorable changes on outcomes, such as weight status and psychosocial variables [47]. We did not ask pairs of children to cooperatively play AVGs to earn the most points throughout the whole intervention. League competitions between pairs of children during the intervention, like presenting weekly league table for incentive, might encourage children to support each other to be more active and positively influence psychosocial variables.

The present study included no component related to family. Therefore, it is not surprising that the AVG intervention had null effect on family social support for PA. Parental support is an important PA determinant of children [53]. Therefore, family related strategies can be combined with future school-based AVG intervention. However, engaging parents in the intervention has been a difficulty to promote children’s PA [54]. Given that PA or exercise is not a valued educational element in the current examination culture in Hong Kong, parental support of PA seems hard to be enhanced. Research assistants instead of classroom teachers organized children’s play in the present study. Eather and colleagues indicated that teachers played a key role in influencing young student’s PA behavior [55]. Teacher could account for approximately 30% of the variance in learning [56]. The powerful influence of teachers should be considered in the future school-based AVG interventions.

No mediation effects of the selected PA-related psychosocial factors were found in the current study. These findings were mainly due to the lack of treatment effects on these PA-related variables. Mediation effects of PA-related psychosocial factors were seldom examined in interventions among primary school children [57]. It may because children were lack of behavior autonomy and cognitive ability in this age group. Thus, other factors may be important for the explanations of the treatment effects of AVG interventions on PA and other relevant outcomes. Maddison and his colleagues found that aerobic fitness mediated the treatment effect of a home-based AVG intervention on BMI [58]. Other PA correlates are warranted to be examined in future AVG trials. No moderation effects of the psychosocial factors were examined as well. The heterogeneity in these psychosocial factors among participants may not be sufficient to examine the moderation effects [23].

Strengths of the study included the use of an objective measure of sedentary time and PA, and the use of measures that have been validated to assess PA-related psychosocial variables. Both school-day PA and after-school PA data were processed and analyzed, providing the detailed information to infer the intervention effect on sedentary time and PA. The major limitation of this study was the use of a quasi-experiment design, which may have affected the internal validity. Furthermore, all of the participants were recruited from the same school, which may have introduced contamination between groups, and limited external validity.

## 5. Conclusions

An eight-week AVG intervention decreased sedentary behavior during targeted after-school time and increased overall PA (mainly in LPA) among Hong Kong Chinese primary school children. The intervention effect on body composition in boys is warranted to be further examined in a more robust design. Study results indicated that a school-based AVG intervention is feasible to let children get active. Future studies should consider using more strategies to combine with AVG play to induce more health benefits in children, and further examine in what circumstances such AVG interventions can influence psychosocial factors. The following suggestions could be considered in the future study among primary school children: (a) examine the process of the sedentary behavior and PA changes during a longer intervention period; (b) combine the AVG play in the school and family settings; (c) examine the effect of teacher’s attitude on children’s PA in the school-based AVG intervention; (d) examine dietary outcomes; (e) employ popular AVG sports in different genders and cultures.

## Figures and Tables

**Table 1:** Baseline Characteristics of All Participants (N = 87).

Baseline Characteristics	Intervention (*n* = 30)	Control (*n* = 57)
Age	10.5 (0.7)	10.4 (0.8)
Male (n [%])	24 (80)	30 (53)
Height (cm)	138.4 (7.0)	141.2 (7.1)
Weight (kg)	35.6 (9.1)	36.7 (9.4)
BMI (kg/m^2^)	18.4 (4.0)	18.2 (3.3)
Daily Sedentary Screen Time (min)	123.8 (130.9)	141.4 (164.7)

Note. Mean (standard deviation) were reported, unless otherwise stated.

**Table 2:** Estimated Treatment Effects on Accelerometer Outcomes.

Outcomes					Change from Baseline Outcome			
	I, Mean (SD)		C, Mean (SD)		I, Mean (SE)	C, Mean (SE)	I-C Difference (95% CI)	*p*-Value
	Baseline	8 Weeks	Baseline	8 Weeks				
Waking Time PA and Sedentary Behavior								
MVPA (min/day)	21.3 (9.6)	22.6 (11.9)	21.4 (10.4)	18.9 (10.2)	1.2 (1.6)	−2.4 (1.3)	3.6 (−0.6, 7.8)	0.12
MPA (min/day)	18.0 (7.9)	19.2 (9.4)	18.4 (8.5)	16.0 (8.0)	1.1 (1.3)	−2.3 (1.0)	3.4 (−0.3, 7.3)	0.06
VPA (min/day)	3.3 (2.3)	3.4 (3.4)	3.0 (2.3)	2.9 (2.6)	0.1 (0.5)	−0.1 (0.4)	0.2 (−1.1, 1.6)	0.86
LPA (min/day)	272.4 (86.3)	278.1 (93.7)	275.6 (65.0)	242.3 (72.2)	3.2 (9.5)	−31.7 (7.7)	34.9 (8.7, 58.2)	0.01
Sedentary Time (min/day)	465.3 (71.9)	439.5 (76.2)	470.4 (73.6)	471.4 (65.8)	−30.1 (12.4)	3.8 (10.0)	−33.9 (−70.8, 4.8)	0.07
CPM	318.6 (80.3)	346.3 (105.3)	309.6 (79.9)	284.4 (80.4)	28.2 (16.6)	−25.5 (13.4)	53.7 (8.6, 104.2)	0.04
After-School Time PA and Sedentary Behavior								
MVPA (min/day)	8.4 (5.7)	8.8 (5.8)	10.5 (7.4)	7.1 (6.7)	0 (1.1)	−3.1 (0.9)	3.0 (0.2, 5.9)	0.07
MPA (min/day)	7.0 (5.1)	7.4 (4.9)	8.9 (6.0)	6.1 (5.8)	−0.1 (1.0)	−2.5 (0.8)	2.4 (−0.2, 5.1)	0.08
VPA (min/day)	1.4 (1.2)	1.4 (1.4)	1.5 (1.6)	1.0 (1.2)	0 (0.2)	−0.5 (0.2)	0.5 (−0.2, 1.2)	0.16
LPA (min/day)	107.8 (60.1)	101.6 (60.7)	113.3 (45.6)	81.0 (47.8)	−7.2 (4.7)	−31.7 (3.8)	25.0 (13.7, 34.5)	<0.01
Sedentary Time (min/day)	182.8 (44.8)	164.9 (40.3)	190.2 (54.0)	187.7 (49.3)	−22.8 (6.3)	0.7 (5.1)	−23.5 (−41.7, −5.4)	0.01
CPM	297.4 (114.1)	349.7 (144.8)	344.8 (132.6)	260.7 (144.8)	36.0 (28.4)	−73.4(22.8)	109.4 (36.4, 178.8)	0.01

Note: Outcome values at baseline and 8 weeks of the participants who were included in the analyses were reported. Mean (SE) of change from baseline outcome were reported, with adjusted group difference, 95% CI and associated p-value based on 1000 bootstrap samples. C, control group; CI, confidence interval; CPM, counts per minute; I, intervention group; LPA, light physical activity; min, minute; MPA, moderate physical activity; MVPA, moderate-to-vigorous physical activity; PA, physical activity; SD, stand deviation; SE, stand error; VPA, vigorous physical activity.

**Table 3:** Estimated Treatment Effects on Body Composition.

Outcomes					Change from Baseline Outcome			
	I, Mean (SD)		C, Mean (SD)		I, Mean (SE)	C, Mean (SE)	I-C Difference (95% CI)	*p*-Value
	Baseline	8 Weeks	Baseline	8 Weeks				
Body Composition								
zBMI	0.4 (1.4)	0.4 (1.3)	0.3 (1.2)	0.4 (1.2)	0 (0)	0 (0)	0 (−0.1, 0.1)	0.42
Percentage body fat (%)	20.6 (8.5)	19.6 (7.5)	19.7 (6.6)	19.1 (6.7)	−0.9 (0.4)	−0.7 (0.3)	−0.2 (−1.5, 0.9)	0.71

Note. Outcome values at baseline and 8 weeks of the participants who were included in the analyses were reported. Mean (SE) of change from baseline outcome were reported, with adjusted group difference, 95% CI and associated *p*-value based on 1000 bootstrap samples. C, control group; CI, confidence interval; I, intervention group; SD, stand deviation; SE, stand error.

**Table 4:** Estimated Treatment Effects on Psychosocial Factors.

Outcomes					Change from Baseline Outcome			
	I, Mean (SD)		C, Mean (SD)		I, Mean (SE)	C, Mean (SE)	I-C Difference (95% CI)	*p*-Value
	Baseline	8 Weeks	Baseline	8 Weeks				
Body Composition								
Enjoyment	1.8 (0.9)	2.4 (1.2)	1.6 (0.8)	2.0 (1.1)	0.6 (0.2)	0.3 (0.2)	0.3 (−0.3, 0.8)	0.39
Self-efficacy	3.7 (0.8)	3.3 (1.0)	3.7 (0.8)	3.6 (1.0)	−0.4 (0.2)	−0.1 (0.1)	−0.3 (−0.8, 0.1)	0.17
SSFR	2.0 (1.0)	2.2 (1.2)	2.2 (1.1)	2.3 (1.3)	0.1 (0.2)	0.2 (0.2)	−0.1 (−0.6, 0.6)	0.85
SSFA	2.6 (1.1)	2.7 (1.2)	2.8 (1.1)	2.5 (1.2)	0.1 (0.2)	−0.2 (0.2)	0.3 (−0.3, 0.9)	0.31

Note. Outcome values at baseline and 8 weeks of the participants who were included in the analyses were reported. Mean (SE) of change from baseline outcome were reported, with adjusted group difference, 95% CI and associated p-value based on 1000 bootstrap samples. C, control group; CI, confidence interval; I, intervention group; SD, stand deviation; SE, stand error.

**Table 5:** Estimated Treatment Effects among Boys.

Outcomes					Change from Baseline Outcome			
	I, Mean (SD)		C, Mean (SD)		I, Mean (SE)	C, Mean (SE)	I-C Difference (95% CI)	*p*-Value
	Baseline	8 Weeks	Baseline	8 Weeks				
**Waking Time PA and Sedentary Behavior**								
MVPA (min/day)	21.9 (9.6)	23.4 (12.2)	24.1 (11.4)	21.0 (11.3)	1.1 (1.9)	−2.8 (1.7)	3.9 (−0.9, 9.2)	0.18
MPA (min/day)	18.7 (7.9)	19.7 (9.3)	20.5 (9.3)	17.5 (8.8)	0.6 (1.5)	−2.6 (1.3)	3.2 (−1.0, 7.6)	0.13
VPA (min/day)	3.2 (2.2)	3.7 (3.6)	3.6 (2.6)	3.5 (2.9)	0.4 (0.6)	−0.1 (0.6)	0.5 (−1.2, 2.4)	0.58
LPA (min/day)	271.7 (93.1)	280.7 (94.5)	283.1 (71.5)	244.0 (80.3)	4.7 (9.9)	−35.7 (9.0)	40.4 (13.3, 64.4)	0.01
Sedentary Time (min/day)	472.7 (68.4)	438.1 (74.4)	459.2 (73.2)	474.1 (55.1)	−30.1 (12.9)	11.2 (11.7)	−41.4 (−79.8, 0.1)	0.04
CPM	313.8 (81.2)	354.4 (107.2)	331.1 (85.6)	295.7 (90.8)	36.3 (19.1)	−31.8 (17.3)	68.1 (21.5, 117.9)	0.02
**After-school Time PA and Sedentary Behavior**								
MVPA (min/day)	9.5 (5.7)	8.5 (5.9)	12.8 (8.3)	8.5 (7.5)	−1.6 (1.3)	−3.8 (1.2)	2.2 (−1.6, 6.2)	0.26
MPA (min/day)	8.0 (5.1)	7.1 (4.9)	10.8 (6.8)	7.2 (6.4)	−1.6 (1.1)	−3.2 (1.0)	1.6 (−1.5, 4.8)	0.31
VPA (min/day)	1.5 (1.2)	1.4 (1.6)	2.0 (1.8)	1.3 (1.4)	−0.2 (0.3)	−0.6 (0.3)	0.5 (−0.3, 1.5)	0.27
LPA (min/day)	117.8 (57.4)	105.4 (64.3)	121.2 (48.7)	86.2 (51.4)	−10.5 (5.2)	−36.3 (4.6)	25.8 (12.3, 37.7)	<0.01
Sedentary Time (min/day)	191.7 (39.4)	166.9 (41.4)	191.2 (54.0)	194.8 (48.5)	−24.5 (6.9)	1.4 (6.1)	−25.9 (−47.3, −4.4)	0.01
CPM	316.4 (100.4)	348.0 (147.9)	373.7 (127.9)	276.9 (167.5)	13.9 (34.6)	−90.5 (30.2)	104.4 (11.0, 194.8)	0.03
**Body Composition**								
zBMI	0.5 (1.4)	0.5 (1.4)	0.4 (1.0)	0.4 (1.0)	0 (0)	0.1 (0)	−0.1 (−0.2, 0)	0.04
Percentage body fat (%)	21.3 (8.6)	19.9 (7.8)	19.7 (5.3)	19.7 (5.8)	−1.3 (0.7)	−0.1 (0.7)	−1.2 (−2.9, 0.5)	0.23
**Psychosocial Factors**								
Enjoyment	1.9 (0.9)	2.3 (1.3)	1.5 (0.6)	2.1 (1.2)	0.7 (0.3)	0.5 (0.2)	0.2 (−0.5, 0.8)	0.67
Self-efficacy	3.7 (0.8)	3.3 (1.0)	3.9 (0.9)	3.8 (1.1)	−0.4 (0.2)	−0.1 (0.2)	−0.4 (−0.9, 0.2)	0.19
SSFR	1.9 (1.0)	2.2 (1.2)	2.2 (1.1)	2.5 (1.4)	0.2 (0.3)	0.4 (0.2)	−0.16 (−0.8, 0.6)	0.60
SSFA	2.5 (1.1)	2.8 (1.3)	2.8 (1.2)	2.4 (1.3)	0.1 (0.3)	−0.3 (0.2)	0.4 (−0.2, 1.1)	0.25

Note: Outcome values at baseline and 8 weeks of the participants who were included in the analyses are reported. Mean (SE) of change from baseline outcome are reported, with adjusted group difference, 95% CI and associated p-value based on 1000 bootstrap samples, and estimated effect size. C, control group; CI, confidence interval; CPM, counts per minute; I, intervention group; LPA, light physical activity; min, minute; MPA, moderate physical activity; MVPA, moderate-to-vigorous physical activity; PA, physical activity; SD, stand deviation; SE, stand error; SSFA, social support from family; SSFR, social support from friends; VPA, vigorous physical activity; zBMI, standardized body mass index.

## References

[1] Marshall S.J., Welk G.J., Smit A.L., Biddle S.J.H. (2008). Definitions and measurement. Youth Physical Activity and Sedentary Behavior: Challenges and Solutions.

[2] World Health Organization (2010). Global Recommendations on Physical Activity for Health. https://www.who.int/dietphysicalactivity/publications/9789241599979/en/.

[3] Tremblay M.S., LeBlanc A.G., Janssen I., Kho M.E., Hicks A., Murumets K., Colley R.C., Duggan M. (2011). Canadian sedentary behaviour guidelines for children and youth. Appl. Physiol. Nutr. Metab..

[4] World Health Organization (2012). Population-Based Approaches to Childhood Obesity Prevention. https://www.who.int/dietphysicalactivity/childhood/WHO_new_childhoodobesity_PREVENTION_27nov_HR_PRINT_OK.pdf.

[5] Census and Statistics Department of Hong Kong Special Administrative Region (2013). Healthy Exercise for All Campaign-Physical Fitness Test for the Community. https://www.statistics.gov.hk/pub/B71302FA2013XXXXB0100.pdf.

[6] Department of Health of Hong Kong Special Administrative Region (2018). Obesity: A Weighty Health Issue Key Messages. https://www.chp.gov.hk/files/pdf/ncd_watch_august_2018.pdf.

[7] Department of Health of Hong Kong Special Administrative Region Statistics. https://www.chp.gov.hk/en/statistics/data/10/757/5513.html.

[8] Sallis J.F., Prochaska J.J., Taylor W.C. (2000). A review of correlates of physical activity of children and adolescents. Med. Sci. Sports Exerc..

[9] Education Department of Hong Kong Special Administrative Region (2000). Physical Space and Learning Environment of Primary and Secondary Schools. https://www.legco.gov.hk/yr99-00/english/panels/ed/papers/e1693-03.pdf.

[10] Salmon J. (2010). Novel strategies to promote children’s physical activities and reduce sedentary behavior. J. Phys. Act. Health.

[11] Liang Y., Lau P.W.C. (2014). Effects of active videogames on physical activity and related outcomes among healthy children: A systematic review. Games Health J..

[12] Norman G.J., Adams M.A., Ramirez E.R., Carlson J.A., Kerr J., Godbole S., Marshall S.J. (2013). Effects of behavioral contingencies on adolescent active videogame play and overall activity: A randomized trial. Games Health J..

[13] Baranowski T., Abdelsamad D., Baranowski J., O’Connor T.M., Thompson D., Barnett A., Cerin E., Chen T.A. (2012). Impact of an active video game on healthy children’s physical activity. Pediatrics.

[14] Graves L.E.F., Ridgers N.D., Atkinson G., Stratton G. (2010). The effect of active video gaming on children’s physical activity, behavior preferences and body composition. Pediatr. Exerc. Sci..

[15] Maddison R., Foley L., Ni Mhurchu C., Jiang Y., Jull A., Prapavessis H., Hohepa M., Rodgers A. (2011). Effects of active video games on body composition: A randomized controlled trial. Am. J. Clin. Nutr..

[16] Maloney A.E., Bethea T.C., Kelsey K.S., Marks J.T., Paez S., Rosenberg A.M., Catellier D.J., Hamer R.M., Sikich L. (2008). A pilot of a video game (DDR) to promote physical activity and decrease sedentary screen time. Obesity.

[17] Maloney A.E., Threlkeld K.A., Cook W.L. (2012). Comparative effectiveness of a 12-week physical activity intervention for overweight and obese youth: Exergaming with “Dance Dance Revolution”. Games Health J..

[18] Mhurchu C.N., Maddison R., Jiang Y., Jull A., Prapavessis H., Rodgers A. (2008). Couch potatoes to jumping beans: A pilot study of the effect of active video games on physical activity in children. Int. J. Behav. Nutr. Phys. Act..

[19] Chin A Paw M.J.M., Jacobs W.M., Vaessen E.P.G., Titze S., van Mechelen W. (2008). The motivation of children to play an active video game. J. Sci. Med. Sport.

[20] Lau P.W.C., Wang J.J., Maddison R. (2016). A randomized controlled trial of school-based activevideo game intervention on Chinese children’s aerobic fitness, physical activity level and psychological correlates. Games Health J..

[21] Gao Z., Huang C., Liu T., Xiong W. (2012). Impact of interactive dance games on urban children’s physical activity correlates and behavior. J. Exerc. Sci. Fit..

[22] Metcalf B., Henley W., Wilkin T. (2012). Effectiveness of intervention on physical activity of children: Systematic review and meta-analysis of controlled trials with objectively measured outcomes. BMJ.

[23] Liang Y. (2015). Effects of Active Video Game Intervention on Promoting Physical Activity among Hong Kong Chinese Children. Ph.D. Thesis.

[24] Reading S.A., Prickett K. (2013). Evaluation of children playing a new-generation motion-sensitive active video game by accelerometry and indirect calorimetry. Games Health J..

[25] Fortier M.S., Sweet S.N., O’Sullivan T.L., Williams G.C. (2007). A self-determination process model of physical activity adoption in the context of a randomized controlled trial. Psychol. Sport Exerc..

[26] Smallwood S.R., Morris M.M., Fallows S.J., Buckley J.P. (2012). Physiologic responses and energy expenditure of Kinect active video game play in schoolchildren. Arch. Pediatr. Adolesc. Med..

[27] Sung R.Y., Lau P., Yu C.W., Lam P.K., Nelson E.A. (2001). Measurement of body fat using leg to leg bioimpedance. Arch. Dis. Child.

[28] Cole T.J., Lobstein T. (2012). Extended international (IOTF) body mass index cut-offs for thinness, overweight and obesity. Pediatr. Obes..

[29] Weintraub D.L., Tirumalai E.C., Haydel K.F., Fujimoto M., Fulton J.E., Robinson T.N. (2008). Team sports for overweight children: The Stanford sports to prevent obesity randomized trial (SPORT). Arch. Pediatr. Adolesc. Med..

[30] Kriemler S., Zahner L., Schindler C., Meyer U., Hartmann T., Hebestreit H., Brunner-La Rocca H.P., van Mechelen W., Puder J.J. (2010). Effect of school based physical activity programme (KISS) on fitness and adiposity in primary schoolchildren: Cluster randomised controlled trial. BMJ.

[31] Evenson K.R., Catellier D.J., Gill K., Ondrak K.S., McMurray R.G. (2008). Calibration of two objective measures of physical activity for children. J. Sports Sci..

[32] Trost S.G., Loprinzi P.D., Moore R., Pfeiffer K.A. (2011). Comparison of accelerometer cut points for predicting activity intensity in youth. Med. Sci. Sports Exerc..

[33] Huang W.Y., Wong S.H., Salmon J. (2013). Correlates of physical activity and screen-based behaviors in Chinese children. J. Sci. Med. Sport.

[34] Ward D.S., Saumders R.P., Pate R.R. (2007). Physical Activity Interventions in Children and Adolescents.

[35] Sallis J.F., Grossman R.M., Pinski R.B., Patterson T.L., Nader P.R. (1987). The development of scales to measure social support for diet and exercise behaviors. Prev. Med..

[36] Liang Y., Lau P.W.C., Huang W.Y.J., Maddison R., Baranowski T. (2014). Validity and reliability of questionnaires measuring physical activity self-efficacy, enjoyment, social support among Hong Kong Chinese children. Prev. Med. Rep..

[37] Motl R.W., Dishman R.K., Trost S.G., Saunders R.P., Dowda M., Felton G., Ward D.S., Pate R.R. (2000). Factorial validity and invariance of questionnaires measuring social-cognitive determinants of physical activity among adolescent girls. Prev. Med..

[38] Kraemer H.C., Wilson G.T., Fairburn C.G., Agras W.S. (2002). Mediators and moderators of treatment effects in randomized clinical trials. Arch. Gen. Psychiatry.

[39] Patnode C.D., Lytle L.A., Erickson D.J., Sirard J.R., Barr-Anderson D., Story M. (2010). The relative influence of demographic, individual, social, and environmental factors on physical activity among boys and girls. Int. J. Behav. Nutr. Phys. Act..

[40] Lindner K.J. (1999). Sport participation and perceived academic performance of school children and youth. Pediatr. Exerc. Sci..

[41] Guo Q., Wang X.Z., Jiang J.B. (2017). The patterns of physical activity and sedentary behavior in Chinese children and adolescents. China Sport Sci..

[42] de Rezende L.F., Rodrigues Lopes M., Rey-López J.P., Matsudo V.K., Luiz O. (2014). Sedentary behavior and health outcomes: An overview of systematic reviews. PLoS ONE.

[43] Carson V., Hunter S., Kuzik N., Gray C.E., Poitras V.J., Chaput J.P., Saunders T.J., Katzmarzyk P.T., Okely A.D., Connor Gorber S. (2016). Systematic review of sedentary behaviour and health indicators in school-aged children and youth: An update. Appl. Physiol. Nutr. Metab..

[44] Kwon S., Janz K.F., Burns T.L., Levy S.M. (2011). Association between light-intensity physical activity and adiposity in childhood. Pediatr. Exerc. Sci..

[45] Poitras V.J., Gray C.E., Borghese M.M., Carson V., Chaput J.P., Janssen I., Katzmarzyk P.T., Pate R.R., Connor Gorber S., Kho M.E. (2016). Systematic review of the relationships between objectively measured physical activity and health indicators in school-aged children and youth. Appl. Physiol. Nutr. Metab..

[46] Shqair A.Q., Pauli L.A., Costa V., Cenci M., Goettems M.L. (2019). Screen time, dietary patterns and intake of potentially cariogenic food in children: A systematic review. J. Dent..

[47] Staiano A.E., Abraham A.A., Calvert S.L. (2013). Adolescent exergame play for weight loss and psychosocial improvement: A controlled physical activity intervention. Obesity.

[48] Trost S.G., Sundal D., Foster G.D., Lent M.R., Vojta D. (2014). Effects of a pediatric weight management program with and without active video games a randomized trial. JAMA Pediatr..

[49] Gao Z., Xiang P. (2014). Effects of exergaming based exercise on urban children’s physical activity participation and body composition. J. Phys. Act. Health.

[50] White K., Schofield G., Kilding A.E. (2011). Energy expended by boys playing active video games. J. Sci. Med. Sport.

[51] Graves L.E.F., Ridgers N.D., Williams K., Stratton G., Atkinson G., Cable N.T. (2010). The physiological cost and enjoyment of Wii Fit in adolescents, young adults, and older adults. J. Phys. Act. Health.

[52] Penko A.L., Barkley J.E. (2010). Motivation and physiologic responses of playing a physically interactive video game relative to a sedentary alternative in children. Ann. Behav. Med..

[53] Lu C., Stolk R.P., Sauer P.J., Sijtsma A., Wiersma R., Huang G., Corpeleijn E. (2017). Factors of physical activity among Chinese children and adolescents: A systematic review. Int. J. Behav. Nutr. Phys. Act..

[54] O’Connor T.M., Jago R., Baranowski T. (2009). Engaging parents to increase youth physical activity a systematic review. Am. J. Prev. Med..

[55] Eather N., Morgan P.J., Lubans D.R. (2013). Social support from teachers mediates physical activity behavior change in children participating in the Fit-4-Fun intervention. Int. J. Behav. Nutr. Phys. Act..

[56] Hattie J.A.C. Teachers make a difference: What is the research evidence?. Proceedings of the Building Teacher Quality: What Does the Research Tell Us ACER Research Conference.

[57] Brown H., Hume C., Pearson N., Salmon J. (2013). A systematic review of intervention effects on potential mediators of children’s physical activity. BMC. Public Health.

[58] Maddison R., Mhurchu C.N., Jull A., Prapavessis H., Foley L.S., Jiang Y. (2012). Active video games: The mediating effect of aerobic fitness on body composition. Int. J. Behav. Nutr. Phys. Act..

